# Plasma Free Fatty Acids Metabolic Profile with LC-MS and Appetite-Related Hormones in South Asian and White European Men in Relation to Adiposity, Physical Activity and Cardiorespiratory Fitness: A Cross-Sectional Study

**DOI:** 10.3390/metabo9040071

**Published:** 2019-04-13

**Authors:** Simone Benedetti, Naser F. Al-Tannak, Mansour Alzharani, Hannah J. Moir, David J. Stensel, Alice E. Thackray, Declan P. Naughton, Mehmet T. Dorak, Owen Spendiff, Natasha Hill, David G. Watson, Judith Allgrove

**Affiliations:** 1 Applied & Human Sciences, School of Life Sciences, Pharmacy & Chemistry, Kingston University London, Kingston upon Thames KT1 2EE, UK; S.Benedetti@kingston.ac.uk (S.B.); H.Moir@kingston.ac.uk (H.J.M.); D.Naughton@kingston.ac.uk (D.P.N.); M.Dorak@kingston.ac.uk (M.T.D.); O.Spendiff@kingston.ac.uk (O.S.); N.Hill@kingston.ac.uk (N.H.); 2 Department of Pharmaceutical Chemistry, Faculty of Pharmacy, Kuwait University, P.O. Box 23924, Safat, Kuwait City 13110, Kuwait; dr_altannak@HSC.EDU.KW; 3 Strathclyde Institute of Pharmacy and Biomedical Sciences, University of Strathclyde, The John Arbuthnott Building, 161 Cathedral Street, Glasgow G4 0RE, UK; mansour.alzahrani@strath.ac.uk; 4 National Centre for Sport and Exercise Medicine, School of Sport, Exercise and Health Sciences, Loughborough University, Loughborough LE11 3TU, UK; D.J.Stensel@lboro.ac.uk (D.J.S.); A.E.Thackray@lboro.ac.uk (A.E.T.); 5 University Hospitals of Leicester NHS Trust, Infirmary Square, Leicester LE1 5WW, UK

**Keywords:** metabolomics, free fatty acids, cardiovascular disease, Type 2 diabetes, South Asian, physical activity, cardiorespiratory fitness, inflammation, appetite hormones, metabolic markers

## Abstract

South Asians have a greater cardiovascular disease (CVD) and type 2 diabetes (T2D) risk than white Europeans, but the mechanisms are poorly understood. This study examined ethnic differences in free fatty acids (FFAs) metabolic profile (assessed using liquid chromatography-mass spectrometry), appetite-related hormones and traditional CVD and T2D risk markers in blood samples collected from 16 South Asian and 16 white European men and explored associations with body composition, objectively-measured physical activity and cardiorespiratory fitness. South Asians exhibited higher concentrations of five FFAs (laurate, myristate, palmitate, linolenic, linoleate; *p* ≤ 0.040), lower acylated ghrelin (ES = 1.00, *p* = 0.008) and higher leptin (ES = 1.11, *p* = 0.004) than white Europeans; total peptide YY was similar between groups (*p* = 0.381). South Asians exhibited elevated fasting insulin, C-reactive protein, interleukin-6, triacylglycerol and ratio of total cholesterol to high-density lipoprotein cholesterol (HDL-C) and lower fasting HDL-C (all ES ≥ 0.74, *p* ≤ 0.053). Controlling for body fat percentage (assessed using air displacement plethysmography) attenuated these differences. Despite similar habitual moderate-to-vigorous physical activity (ES = 0.18, *p* = 0.675), $\dot{V}$O_2max_ was lower in South Asians (ES = 1.36, *p* = 0.001). Circulating FFAs in South Asians were positively correlated with body fat percentage (*r*^2^ = 0.92), body mass (*r*^2^ = 0.86) and AUC glucose (*r*^2^ = 0.89) whereas in white Europeans FFAs were negatively correlated with total step counts (*r*^2^ = 0.96). In conclusion, South Asians exhibited a different FFA profile, lower ghrelin, higher leptin, impaired CVD and T2D risk markers and lower cardiorespiratory fitness than white Europeans.

## 1. Introduction

South Asian individuals originate from the Indian sub-continent and collectively comprise a quarter of the entire world’s population. Many South Asians have migrated to European and North American countries with a large representation living in the United Kingdom (UK) where they are the largest ethnic minority group (~3 million, 4.9% of the population) [1]. South Asians who have migrated to Western nations, as well as those living in the Indian subcontinent, exhibit a greater risk of cardiovascular disease (CVD) and type 2 diabetes (T2D) than their western counterparts [2,3,4]. Furthermore, CVD and T2D manifest 5–10 years earlier, at a lower body mass index (BMI), and are associated with premature complications and mortality in South Asian than white European individuals [2,3]. 

The elevated risk of CVD and T2D in South Asians has been linked to the higher prevalence of insulin resistance and associated CVD risk factors including differences in adiposity, as well as markers of inflammation and metabolic health [3,5]. Specifically, South Asians have a greater percent body fat and accumulation of visceral adipose tissue for a given BMI than white Europeans [6]. Furthermore, compared with other ethnic groups, South Asians are more insulin resistant, glucose intolerant, dyslipidaemic and exhibit a less favourable inflammatory profile including higher levels of C-reactive protein (CRP) and interleukin-6 (IL-6) [3,7]. However, these traditional risk markers do not exclusively explain the higher prevalence of CVD and T2D in the South Asian population and the mechanisms underlying progression to T2D remain poorly understood [8]. Therefore, profiling a greater array of parameters may provide a more holistic insight into cardio-metabolic health outcomes in South Asian and white European individuals and may contribute to identify South Asians at higher risk prior to the onset of T2D and CVD.

Elevated circulating free fatty acids (FFAs) play a central role in liver and skeletal muscle insulin resistance and may contribute to β-cell dysfunction [9,10,11]. Previous evidence reported higher plasma FFA concentrations in individuals with T2D and obesity compared with individuals who are healthy and lean [12,13]. Furthermore, a cross-sectional study conducted in young Canadian adults identified significant positive associations between total fatty acids and markers of insulin resistance in Caucasian and East Asian, but not South Asian, individuals [14]. However, it remains unknown whether circulating FFA concentrations are different between South Asians and white Europeans which may help to improve understanding of the elevated CVD and T2D risk in South Asians.

A well-established postulation for the increased CVD and T2D risk in South Asians is their higher levels of body fatness which may be linked with differences in appetite between South Asian and other ethnicites. Several appetite-related hormones have been implicated in the short-term regulation of food intake, including acylated ghrelin and peptide YY (PYY) which exert orexigenic and anorexigenic effects, respectively [15]. However, it is not known whether circulating acylated ghrelin and PYY concentrations are different between South Asian and white European individuals. Previous evidence has identified ethnic differences in circulating adipokines with individuals of South Asian descent exhibiting elevated leptin concentrations compared with white European individuals [16]. Leptin circulates at concentrations proportional to body fatness [17] and plays a central role in regulating long-term changes in energy homeostasis and body fat.

Physical inactivity is estimated to explain >20% of the excess coronary heart disease (CHD) mortality in UK South Asians after adjustment for potential confounding factors such as socioeconomic status, smoking, diabetes and existing CVD [18]. Low levels of physical activity and cardiorespiratory fitness amongst South Asians are likely to contribute to exacerbating the excess insulin resistance and CVD risk in this population [19,20]. Previous observational evidence suggests that a higher level of self-reported physical activity is associated with a lower risk of CVD and T2D in South Asian individuals [21,22]. However, there is limited evidence examining the relationship of objectively-measured physical activity and cardiorespiratory fitness with plasma FFAs and appetite-related hormones in South Asians.

Therefore, the aim of this study was to investigate ethnic differences in the FFA metabolic profile based on liquid chromatography–mass spectrometry (LC-MS), appetite-related hormones and a variety of traditional risk markers for CVD and T2D in South Asian compared with white European men. In addition, this study aimed to quantify objectively the levels of physical activity and cardiorespiratory fitness in South Asian and white European men and to examine relationships with FFAs, appetite-related hormones and risk markers for CVD and T2D.

## 2. Results

### 2.1. Participant Characteristics

The physical and physiological characteristics of the South Asian and white European participants are shown in Table 1. There were no significant differences between groups in stature, body mass, BMI, waist circumference, resting systolic blood pressure and resting diastolic blood pressure (all *p* ≥ 0.172). Compared with white European participants, South Asian participants exhibited higher fat mass (ES = 0.66, *p* = 0.071) and body fat percentage (ES = 0.86, *p* = 0.021). Fat-free mass (ES = 1.29, *p* = 0.001), age (ES = 0.72, *p* = 0.052) and $\dot{V}$O_2max_ expressed in absolute (ES = 1.79, *p* < 0.001) and relative (ES = 1.36, *p* = 0.001) terms were lower in South Asian compared with white European participants.

### 2.2. Free Fatty Acids Metabolic Profile

Figure S1 shows a comparison between the fatty acid standard mixture and the fatty acids present in a plasma sample. Out of the 37 fatty acids present in the standard, 19 of these could be detected in plasma and quantitative values are given in Table 2. The *p* values for the FFAs were validated using correction for multiple comparisons [23] which indicated for the number of variables used that all *p* values < 0.05 could be regarded as signficant. Linoleic and oleic acid were, by some way, the most abundant FFAs in plasma.

**Figure 1:**
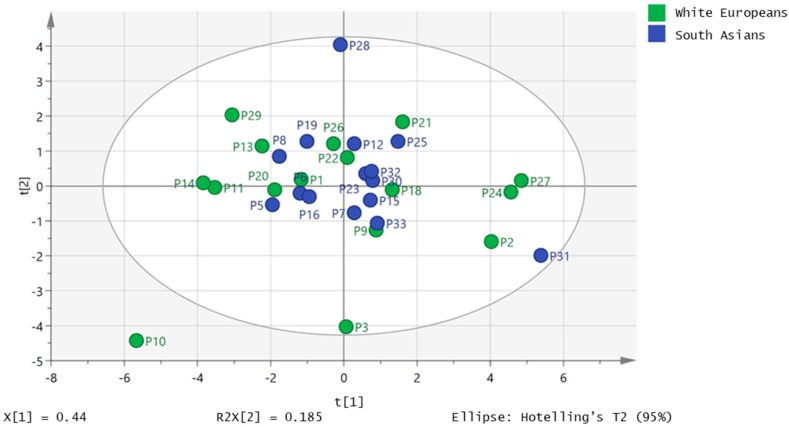
Principle components analysis (PCA) plot for South Asian (*n* = 16) and white European (*n* = 16) samples based on peak quantities of 19 fatty acids in plasma.

**Figure 2:**
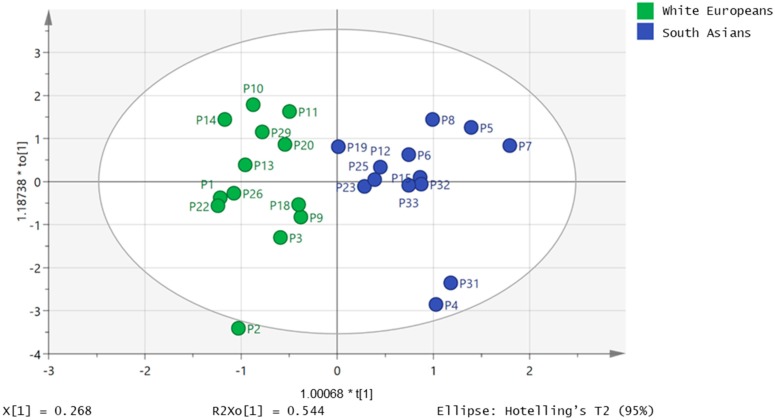
Orthogonal partial least squares (OPLS-DA) plot showing separation of 13 South Asian samples from 13 white European samples based on the concentrations for four fatty acids (myristate, linoleate, linolenate and docosapentenoate) in plasma.

Figure 1 shows the principal components analysis (PCA) plot for the 32 samples gave no differentiation between the white European and South Asian samples. A strong orthogonal partial least squares discriminant analysis (OPLS-DA) model (CVANOVA 5.9 × 10^−7^) could be built which differentiated 13 of the South Asian samples from 13 of the white European samples based on four of the FFAs (Figure 2). 

The cross-validation plot for this model can be seen in Figure S2 and the loadings plot in Figure S3. The loadings are mainly towards the South Asian group and apart from docosapentenoic acid all the FFAs in the model (myristate, linolenic and linoleate) are higher or significantly higher in the South Asian group (Table 1).

**Figure 3:**
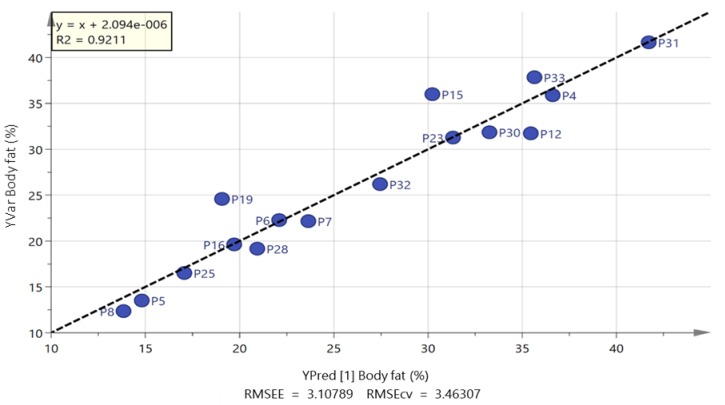
OPLS plot of predicted against actual body fat percentage based on five free fatty acids for South Asian men (palmitate, stearate, linoleate, eicosatetraenoic acid and docosahexaenoic acid).

**Figure 4:**
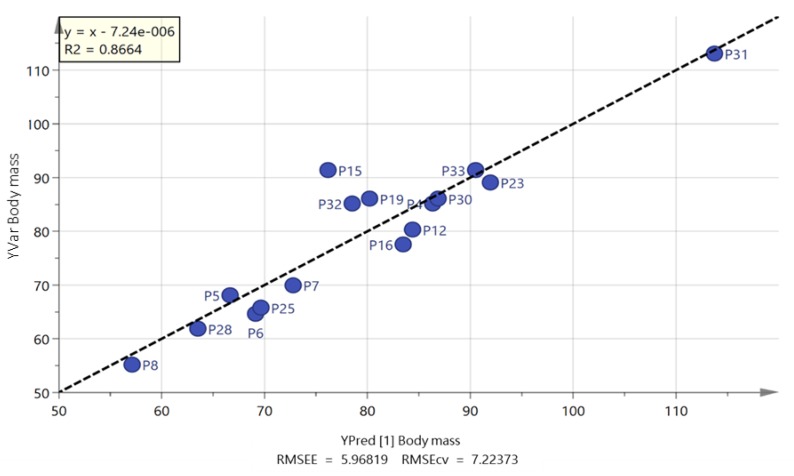
OPLS of predicted vs. actual body mass for South Asian men based on three free fatty acids lignoceric, docosahexenoic and stearic acid.

**Figure 5:**
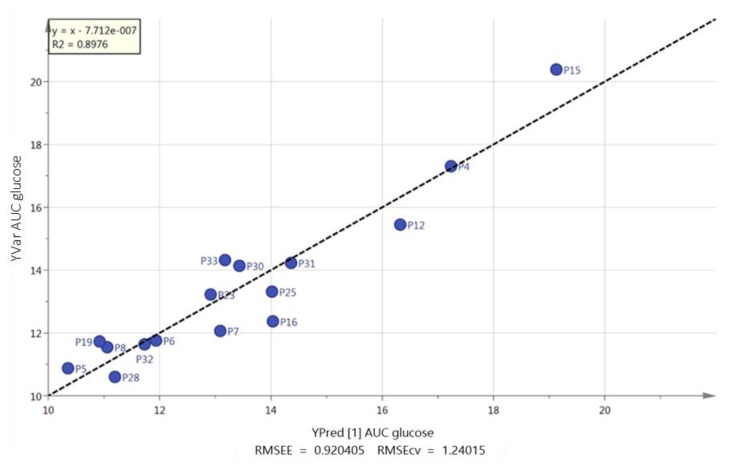
OPLS of predicted vs. actual area under the curve (AUC) for glucose based on four free fatty acids palmitate, oleate, eicosatrienoic acid and docosahexenoic acid.

It was not possible to produce strong orthogonal partial least squares (OPLS) models when trying to fit the data shown in Table 1 to the combined white European and South Asian groups. In addition, when the white European group was modelled in isolation, it was not possible to produce a strong model for any of the parameters in Table 1. However, when the South Asian group was modelled on its own, valid models could be produced for body mass, BMI, AUC glucose and body fat percentage (Figure 3, Figure 4, Figure 5, and Figure S4). The strongest correlation was produced for body fat percentage (*r*^2^ = 0.92; Figure 3) where the loadings indicated that the highest body fat percentage correlated with the highest levels of palmitic, stearic, linoleic, docosapentaenoic and eicosatetraenoic acids (Figure S5). Figure S6 shows extracted ion traces for palmitic and eicosatetraenoic acids for participants P31 and P8 (both South Asians) who had the highest and lowest body fat percentage, respectively. Body mass could also be modelled using an OPLS model and just three FFAs to produce a strong model (*r*^2^ = 0.86; Figure 4). Two of the FFAs, docosahexenoic acid and stearic acid, which correlated with body fat percentage were also used in the OPLS-DA model (Figure 2) for actual against predicted body mass and in addition the long chain fatty acid lignoceric acid was included. In addition, for the South Asian group it was also possible to correlate AUC glucose to four FFAs (palmitate, oleate, eicosatrienoic and docosahexenoic acid) (*r*^2^ = 0.89; Figure 5). It was also, perhaps not surprisingly, possible to fit an OPLS model for predicted BMI against actual BMI using four FFAs; stearate, palmitate, myristate and arachidate (Figure S4) although the correlation (*r*^2^ = 0.79) between predicted and actual BMI was weaker than for OPLS plots for body fat percentage and body mass. It was not possible to produce a strong OPLS model predicting $\dot{V}$O_2max_, or systolic and diastolic blood pressure for the South Asian or white European group. PLS models could also be fitted to the variables used for modelling body fat percentage, body mass and AUC glucose, but the fit was not quite as strong as for the OPLS models.

### 2.3. Fasting Plasma Concentrations of Appetite-Related Hormones, Inflammatory Markers and Lipid Parameters

Fasting plasma concentrations of appetite-related hormones, inflammatory markers and lipid parameters are shown in Table 3. Linear mixed models revealed higher fasting plasma concentrations in the South Asian than white European participants for CRP (113%, ES = 0.87, *p* = 0.019), leptin (187%, ES = 1.11, *p* = 0.004), ratio of total cholesterol to high-density lipoprotein cholesterol (TC/HDL-C) (22%, ES = 0.80, *p* = 0.030) and triacylglycerol (TAG) (43%, ES = 0.74, *p* = 0.044). Compared with white European participants, South Asian participants exhibited lower concentrations of fasting acylated ghrelin (−47%, ES = 1.00, *p* = 0.008) and high-density lipoprotein cholesterol (HDL-C) (−17%, ES = 0.78, *p* = 0.035). Fasting plasma concentrations of IL-6 were meaningfully, albeit not significantly, higher in South Asian than white European participants (57%, ES = 0.86, *p* = 0.074). No between-group differences were seen in fasting plasma total PYY, total cholesterol (TC) or low-density lipoprotein cholesterol (LDL-C) concentrations (all *p* ≥ 0.215). Between-group differences in fasting plasma constituents were attenuated after adjustment for body fat percentage (*p* ≥ 0.080), although a tendency for a higher leptin concentration in South Asians remained (37%; ES = 0.33, *p* = 0.061).

### 2.4. Plasma Glucose and Insulin Concentrations during the OGTT

Plasma glucose concentrations in the fasted state and 2 h post-challenge were not significantly different between South Asian and white European participants in the unadjusted or body fat adjusted models (all *p* ≥ 0.190) (Table 4). South Asian participants exhibited higher insulin concentrations in the fasted state (71%; ES = 1.06, *p* = 0.053) and at 2 h post-challenge (303%; ES = 1.28, *p* = 0.022) (Table 4). Between-group differences in fasting (34%; ES = 0.58, *p* = 0.218) and 2 h post-challenge (110%; ES = 0.68, *p* = 0.082) insulin were diminished after controlling for body fat percentage (Table 4). The homeostasis model assessment of insulin resistance (HOMA-IR) was meaningfully, albeit not significantly, higher in the South Asian compared with white European participants (66%; ES = 0.94, *p* = 0.081) (Table 4). The insulin sensitivity index was lower in the South Asian than white European participants in the unadjusted (61%; ES = 1.22, *p* = 0.016) and body fat adjusted (41%; ES = 0.68, *p* = 0.055) models (Table 4).

**Figure 6:**
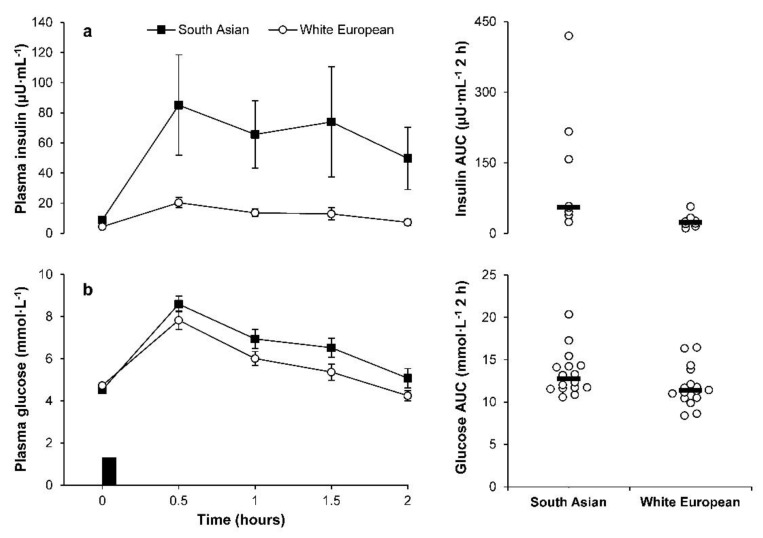
Concentrations of (**a**) insulin (top panels; South Asian *n* = 8, white European *n* = 8) and (**b**) glucose (bottom panels; South Asian *n* = 16; white European *n* = 16) during the oral glucose tolerance test. Data points on left panels represent mean (SEM) for the South Asian (■) and white European (○) men. The black rectangle indicates consumption of glucose load. Data points on the right panels represent individual data values (○) and the solid line indicates the median (▬).

Linear mixed models for glucose OGTT identified a main effect of time (*p* < 0.001) and group-by-time interaction (*p* = 0.047) but not a main effect of group (95% CI −2–26%, *p* = 0.086) (Figure 6). Post-hoc analysis of the group-by-time interaction revealed higher glucose concentrations in the South Asian compared with white European participants at 1.5 h (22%; 95% CI 4–44%, ES = 0.77, *p* = 0.014). The total area under the curve for glucose was higher in the South Asian compared with white European participants (14%; 95% CI −1–30%, ES = 0.69, *p* = 0.060), but this difference was attenuated after adjusting for body fat percentage (5%; 95% CI –8–20%, ES = 0.27, *p* = 0.434) (Figure 6). 

Linear mixed models for insulin OGTT identified a main effect of group (*p* = 0.010), time (*p* < 0.001) and a group-by-time interaction (*p* = 0.025) (Figure 6). The main effect of group revealed higher insulin concentrations in the South Asian compared with white European participants (211%; 95% CI 38–602%, ES = 1.11). The ethnic group difference was diminished but remained significant after adjustment for body fat percentage (100%; 95% CI 8–269%, ES = 0.68, *p* = 0.030). Post hoc analysis of the group-by-time interaction revealed higher insulin concentrations in the South Asian than white European participants at 0.5, 1, 1.5 and 2 h (all ES ≥ 1.28, *p* ≤ 0.013). The total area under the curve for insulin was higher in the South Asian than white European participants in unadjusted (245%; 95% CI 51–690%, ES = 1.61, *p* = 0.006) and body fat adjusted (123%; 95% CI 17–326%, ES = 1.04, *p* = 0.019) models (Figure 6).

### 2.5. Habitual Physical Activity and Sedentary Time

Habitual physical activity levels and sedentary time in the South Asian and white European participants are displayed in Table S1. Three South Asians and three white Europeans did not meet the minimum wear time criteria of at least 10 h per day and were, therefore, excluded from the analysis. No significant differences were seen between the groups for wear time adjusted sedentary time, light activity or MVPA (all *p* ≥ 0.169). Wear time adjusted average CPM (ES = 0.65, *p* = 0.109) and total step counts (ES = 0.71, *p* = 0.142) were meaningfully, albeit not significantly, lower in the South Asian compared with white European participants. 

### 2.6. Dietary Intake

Average protein intake tended to be lower in South Asian compared with white European participants (ES = 0.64, *p* = 0.079), resulting in a lower contribution of protein (ES = 0.70, *p* = 0.064) and a higher contribution of carbohydrate (ES = 0.65, *p* = 0.069) to total energy intake in the South Asian men (Table S2). No other significant differences in energy, macronutrient or micronutrient intakes were observed between the South Asian and white European participants (all *p* ≥ 0.083) (Table S2).

### 2.7. Correlations

**Figure 7:**
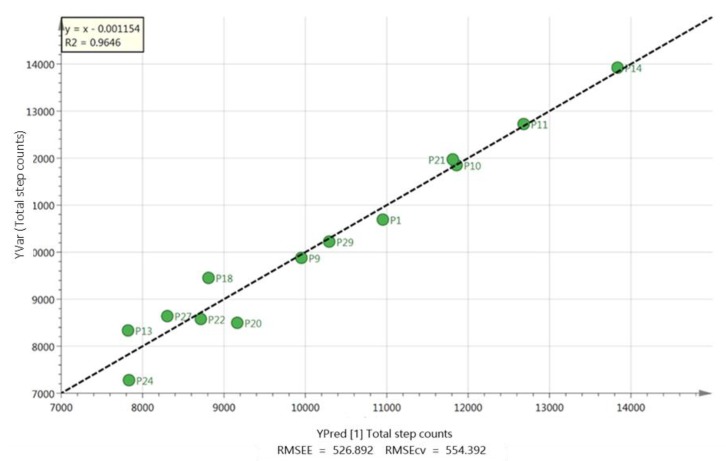
Correlation between total step counts and fatty acids for white European participants based on six fatty acids, palmitoleic, linoleic, arachidate, laurate, erucate and nervonate.

Body fat percentage was positively associated with leptin (*r* = 0.84 to 0.88, *p* ≤ 0.001), and negatively associated with insulin sensitivity index in South Asian and white European men (*r* = −0.76 to −0.83, *p* ≤ 0.047) (Table S3). Body fat percentage was negatively associated with acylated ghrelin (*r* = −0.73, *p* = 0.004) and HDL-C (*r* = −0.81, *p* = 0.001) in white European men only (Table S3). A positive association was also identified between $\dot{V}$O_2max_ and insulin sensitivity index in white European men (*r* = 0.80, *p* = 0.031) (Table S3). In white European but not in South Asian men there was a strong correlation between total step counts and fatty acid profile (*r*^2^ = 0.96; Figure 7) based on six FFAs: palmitoleic, linoleic, arachidate, laurate, erucate and nervonate (Figure S7). Lower step counts were also associated with elevated levels of FFAs, particularly palmitoleic and linoleic acids (Figure S8).

## 3. Discussion

The novel findings of this study are that healthy South Asian men demonstrated higher levels of five FFAs (laurate, myristate, palmitate, linolenic, linoleate) and exhibited lower fasting acylated ghrelin concentrations compared with BMI-matched white European men. Furthermore, South Asian men exhibited impaired CVD and T2D risk markers compared with white European men comprising: (1) elevated fasting concentrations of insulin, leptin, CRP, IL-6 and TAG and a higher TC/HDL-C ratio; (2) lower fasting concentrations of HDL-C; and (3) higher glucose and insulin concentrations during the OGTT. A further key finding is that cardiorespiratory fitness was substantially lower in the South Asian men than in the white European men, but no differences between the ethnicities was observed in objective levels of physical activity or sedentary behaviour. 

In this study, we conducted a cross-sectional analysis to determine whether the FFA metabolic profile varied between healthy South Asian men compared with white European men. We could separate most of the South Asian men from most of the white European men using a strong OPLS-DA model, which provided some confidence that these participants had markedly different fatty acid profiles. Furthermore, it was possible to fit OPLS models for the South Asian men to predict body mass, BMI, AUC glucose and body fat percentage whereas the same models were not a good fit for the white European men. The lack of fit with the data for the white European group lends some additional confidence that the data was not over-fitted which is possible when the sample size is low. 

To the authors’ knowledge, only one previous study has examined ethnic-specific associations between individual plasma fatty acids and markers of insulin resistance in South Asian and European individuals [14]. The previous study looked at total plasma fatty acids by carrying out hydrolysis of plasma TAG post-extraction and this ignores the fact the rate of lipolysis of triglycerides, which is controlled by glucocorticoids and catecholamines, might be important [24]. The present study is the first to focus on ethnic differences in the FFA metabolic profile between healthy South Asian and white European men and explored associations with physical activity and cardiorespiratory fitness levels, in addition to other T2D and CVD risk factors. However, it was not possible to fit OPLS models for the white European individuals predicting body fat percentage, body mass or BMI whereas these models fitted well for the South Asian men. As indicated above, release of fatty acids from adipocytes is under the control of glucorticoids and it might be that there are differences in either glucocorticoid concentration or glucocorticoid sensitivity within the two populations, although it has been found that cortisol levels are actually lower in South Asians in comparison with white Europeans [25]. There were only five FFAs which were significantly different between white European and South Asian men; lauric, myristic, palmitic, linolenic and linoleic. These were all significantly higher in the South Asian group. 

However, the major difference in the two groups was in the correlation of FFAs with body fat percentage and body mass in the South Asian group and between fatty acids and physical activity in the white European group. In the South Asian group higher levels of FFAs were positively correlated with body fat percentage (Figure 3). Higher levels of FFAs have been correlated with both obesity and insulin resistance [12,26]; thus, it may be that in the South Asian group higher body fat percentage resulted in higher levels of FFAs, but not in the white European group. Previous research found that in response to a five-day high fat diet, South Asians were less responsive to the perilipin (PLIN-5) protein which regulates basal lipolysis which is carried out by adipose triacylglycerol lipase (ATGL) compared with white European individuals. This led to the South Asian group exhibiting higher insulin resistance [27]. The major lipolytic enzyme in the body is hormone sensitive lipase (HSL) which is regulated by catecholamine release whereas ATGL is not under the same hormonal control [28]. In the present study, it is possible that higher levels of ATGL in the South Asian group could result in higher levels of FFAs linked to body mass rather than physical activity. The correlation between total step counts and lower levels of FFAs in the white European group might reflect the fact that the FFAs release is promoted more by HSL, which responds to catecholamine release, rather than by ATGL. Catecholamines also promote peroxisome proliferation [29] which could result in lower levels of FFAs correlating with higher total step counts.

South Asians demonstrated a lower fasting acylated ghrelin concentration than white European men. Although the reason for this finding is unclear, it may be linked to the ethnic group differences in adiposity. Previous research has demonstrated that individuals with obesity exhibit lower circulating concentrations of fasting ghrelin than lean individuals [30]. Although our findings only revealed a large inverse correlation between body fat percentage and acylated ghrelin in the white European men, the ethnic group difference in fasting acylated ghrelin concentrations was mitigated after controlling for body fat percentage. Therefore, it seems plausible that the lower acylated ghrelin concentration in the South Asian men may be linked to the higher body fat levels but further work is required to investigate this.

Similar to previous findings, circulating concentrations of plasma leptin were significantly elevated in the South Asian compared with white European men [16,31]. Plasma leptin concentrations were positively associated with body fat percentage and the elevated leptin levels in South Asian men could be, at least partly, mediated by differences in body fat supporting previous findings in South Asian individuals [17,32]. However, South Asian individuals have also been shown to exhibit higher concentrations of leptin than white European individuals despite a similar body fat percentage [31], and the between-group difference in leptin concentrations in this study was diminished, but not eliminated completely, after controlling for body fat percentage. Consequently, it is possible that irregularities in adipose tissue metabolism concomitant with insulin resistance may contribute to the elevated CVD and T2D risk in South Asians. Despite the between-group differences in acylated ghrelin and leptin, the current study did not find any significant differences in plasma total PYY between South Asian and white European participants.

In support of previous findings, higher fasting insulin and elevated glucose and insulin OGTT concentrations were also observed in South Asian participants compared with white European men [32,33] which are indicative of a greater degree of insulin resistance. These differences are further supported by the higher HOMA-IR and lower insulin sensitivity index observed in the South Asian participants. It is well established that insulin resistance is a primary determinant of the elevated propensity for CVD and T2D in individuals of South Asian descent [2,3,7]. One factor suggested to contribute to the excess insulin resistance in South Asian individuals represents differences in adiposity and body fat distribution [2]. In accord with our findings, South Asian individuals exhibit a higher body fat percentage and lower lean body mass for a given BMI than white European individuals [6]. The present study also measured circulating concentrations of fasting IL-6 and CRP which represent key indicators of chronic low-grade inflammation and have been implicated in explaining the excess CHD risk in South Asian individuals [7]. The elevated fasting IL-6 and CRP concentration in South Asian compared with white European men supports several previous studies [34,35], although this finding is not universal [32]. Considering pro-inflammatory IL-6 released from adipose tissue has been identified as a precursor for hepatic CRP secretion [35], it is possible that the ethnic differences in inflammation may be mediated by the higher body fat levels in South Asian individuals. However, future work is required to determine the independent contribution of ethnicity and adiposity on inflammatory markers in South Asians. 

Consistent with previous studies [34,35,36], the South Asian men exhibited a less favourable fasted lipid profile than the white European men encompassing lower concentrations of HDL-C coupled with an elevated TC/HDL-C ratio and higher TAG concentration. The reasons for the adverse lipid profile in South Asian participants have not been fully elucidated, but it is proposed that the greater insulin resistance experienced by South Asians may be implicated [37]. Regardless of the mechanism, our findings contribute to existing knowledge regarding the differential lipid profiles between individuals of South Asian and white European descent.

Previous research suggests that South Asian individuals engage in less habitual physical activity than white European individuals, which is likely to contribute to the excess CHD and T2D risk in this population [18,19,38]. The existing evidence on habitual physical activity levels in South Asians has largely been gleaned from self-report questionnaires [19,20], but data using accelerometry is emerging [38,39] and the objective accelerometer measurement represents a strength of this study. Although the similar levels of MVPA and sedentary time between the ethnicities in this study appears to contradict the aforementioned studies, the South Asian participants accumulated less total activity (CPM) and fewer steps, and stark differences in CVD and T2D risk markers were still apparent between the ethnic groups. This is in line with previous evidence suggesting that South Asian individuals are more insulin resistant than white European individuals even after adjustment for habitual physical activity levels [38]. Furthermore, it has been suggested recently that South Asians may need to accumulate higher levels of moderate-intensity physical activity equating to an additional 10–15 min per day to achieve a comparable CHD risk factor profile of white Europeans who are meeting the current physical activity recommendations [40].

Despite the similar levels of habitual physical activity between the ethnicities, cardiorespiratory fitness assessed by $\dot{V}$O_2max_ was markedly lower in the South Asian than white European participants. This corroborates previous findings [35,37,41], and there is further evidence that the lower $\dot{V}$O_2max_ in South Asian individuals is independent of physical activity levels [38]. Given the importance of physical activity as a method of enhancing $\dot{V}$O_2max_, these findings add further weight to the proposition that South Asians may need to engage in greater physical activity levels than white Europeans to optimise health outcomes [39]. In addition, it has been demonstrated that low $\dot{V}$O_2max_ was the strongest predictor of the excess insulin resistance seen in UK South Asian compared with white European men [38], although our findings only revealed a positive association between $\dot{V}$O_2max_ and insulin sensitivity index in the white European men. Nevertheless, it is likely that the lower $\dot{V}$O_2max_ in the South Asian individuals may contribute to the heightened cardio-metabolic health risk in this population considering that low cardiorespiratory fitness is a well-established and strong predictor of all-cause mortality and CVD events [42].

A further important consideration in the context of chronic disease risk concerns dietary intake. Whilst the current study identified lower protein intake in the South Asian men, the assessment of dietary intake using self-report represents a limitation due to issues of participant recall bias which makes it difficult to accurately correspond self-reported intake with actual intake [43]. Future work is, therefore, required using more direct and objective measures of dietary intake before definitive conclusions can be drawn.

A limitation of this study concerns the potentially confounding effects of body fat percentage which may have accentuated the differences in CVD and T2D risk markers between the ethnicities. Although South Asians are known to exhibit a higher body fat percentage for a given BMI [6], further research is needed to clarify the role of adiposity and ethnicity in modulating CVD and T2D risk in South Asians. Furthermore, the number of participants in the study was small and the South Asian and white European men were not matched for age. However, our findings revealed marked differences in CVD and T2D risk markers between the ethnicities despite the South Asian men being, on average, six years younger than the white European men, and age was not significantly associated with any of the outcome variables in either ethnic group. Finally, the population sample was mostly limited to South Asian men originating from India and, therefore, further investigations are required in other South Asian groups (e.g., Bangladeshis, Sri Lankan and Bhutanese) and in South Asian women.

## 4. Materials and Methods

### 4.1. Chemicals and Solvents

High-performance liquid chromatography (HPLC) grade acetonitrile (ACN), water, acetic acid and hexane were obtained from Fisher Scientific (Leicestershire, UK). A mixture of fatty acid methyl ester standards (Supelco 37-component fatty acid methyl ester mix) was obtained from Sigma Aldrich (Dorset, UK). The methyl esters were hydrolysed with 1 M KOH by heating at 60 °C for 15 min, the mixture was acidified and extracted into hexane. The hexane stock solution was diluted to the levels required for the calibration curves with ethanol. 31H2-palmitic acid which was used as an internal standard was obtained from Sigma Aldrich (Dorset, UK).

### 4.2. Participants and Experimental Design

A total of 16 South Asian and 16 white European men aged 19–50 years volunteered to participate in this study. The study was approved by Kingston University’s Faculty Ethics Committee and written informed consent was provided by participants before the study commenced. The sample size was calculated using G*Power [44]. Based on previous data [45], it was estimated that a sample size of 16 participants per group would have 89% power at the 0.05 level to detect a between-group difference in fasting leptin of 1.05 between-subject SDs. The South Asian group comprised of eight British Asians born in the UK (UK Indian *n* = 5; UK Pakistani *n* = 1; UK Sri Lankan *n* = 2) and of eight individuals born in South Asia (India *n* = 5; Pakistan *n* = 1; Sri Lanka *n* = 1; Nepal *n* = 1). Conversely, the white European group comprised of nine British born participants and of seven individuals originating from European countries. Groups were matched for BMI. All participants were non-smokers, had no personal history of cardiometabolic disease, and were not taking any anticoagulant or anti-inflammatory medication. Participants completed the Physical Activity Readiness Questionnaire [46] to screen for possible contraindications to exercise.

Using a cross-sectional observational design, participants attended the laboratory on two occasions separated by an interval of 7 to 14 days. Participants were asked to avoid strenuous exercise and not to consume caffeine or alcohol in the 24 h period prior to visits 1 and 2. Participants also consumed 500 mL of plain water the night before visit 2 to ensure euhydration before the exercise test.

### 4.3. Visit 1

#### 4.3.1. Anthropometry and Blood Pressure

Participants arrived at the laboratory between 08:00 and 09:00 after a 9 h overnight fast and completed a 4 h trial. Body mass was measured to the nearest 0.1 kg using a digital scale (Seca Ltd., Hamburg, Germany), and stature was measured to the nearest 0.1 cm using a portable stadiometer (Seca Ltd., Birmingham, UK). Body mass index (BMI) was subsequently calculated as mass (kg) divided by stature squared (m^2^). Waist circumference was measured in duplicate to the nearest 0.1 cm at the midpoint between the xiphoid process and the iliac crest using a standard anthropometric measuring tape (HaB International Ltd., Southam, UK), and the mean of the two measurements was recorded. Body composition was assessed using air displacement plethysmography (BodPod; software version 5.2.0, COSMED, Rome, Italy). After 5 min of seated rest, blood pressure was measured using a digital monitor (Omron M10-IT, Omron Healthcare Co. Ltd., Kyoto, Japan).

#### 4.3.2. Fasting Metabolic Assessment and Oral Glucose Tolerance Test

After completion of the anthropometric and blood pressure measurements, a fasting venous blood sample was obtained from the antecubital vein by a trained phlebotomist for the measurement of appetite-related hormones, inflammatory markers and lipid profiling. A fasting fingertip capillary blood sample was taken to determine insulin and glucose concentrations. Participants then consumed a 75 g glucose load (100% dextrose, BulkPowderTM, Colchester, UK) dissolved in 300 mL of water, marking the start of the oral glucose tolerance test (OGTT). Subsequent fingertip capillary blood samples were collected every 30 min for two hours (0.5, 1, 1.5 and 2 h) to quantify glucose and insulin concentrations whilst participants rested in the semi-supine position.

### 4.4. Visit 2

#### Maximum Oxygen Uptake Test

After a 3 h fast, participants performed an incremental exercise test to volitional exhaustion on an electromagnetically braked cycle ergometer (Lode Excalibur Sport, Groningen, The Netherlands) for the determination of maximum oxygen consumption ($\dot{V}$O_2max_). Participants cycled at a self-selected pedal rate between 70 to 90 revolutions per minute for 3 min at 50 watts (warm up), followed by increments of 6 watts every 15 s until volitional fatigue. Expired air samples were monitored continuously using an online breath-by-breath gas analysis system (Oxycon Pro, Viasys Healthcare Gmbh, Höchberg, Germany). An average of the breath-by-breath $\dot{V}$O_2max_ data was calculated every 15 s, and $\dot{V}$O_2max_ was recorded as the highest 15 s average.

### 4.5. Habitual Physical Activity and Sedentary Time

Between visits 1 and 2, participants wore an ActiGraph GT3X+ accelerometer (ActiGraph, Pensacola, FL, USA) on the right hip for seven consecutive days during waking hours (except water-based activities). All devices were initialised to record counts and steps and raw data files were analysed using the manufacturer’s software (ActiLife v6.2; ActiGraph, Pensacola, FL, USA). Data from participants with at least 10 h of daily wear time for at least four days were included in the analysis. A 60 s sampling epoch was used throughout and non-wear time, defined as ≥60 min of consecutive zero counts, was removed from the analysis [20]. Physical activity was expressed as average CPM and standard cut-points for adults were applied to quantify sedentary time (<100 CPM), light activity (100–1951 CPM) and moderate-to-vigorous activity (>1951 CPM) [47].

### 4.6. Dietary Intake

Participants weighed and recorded their dietary intake on three consecutive days including two weekdays and one weekend day. Participants were also asked to take a digital photograph of all food and drink items consumed during the three-day assessment period which were matched to the diet record. Three-day diet records were analysed using Dietplan 6 software (Forestfield Software Ltd., Horsham, UK).

### 4.7. Blood Sampling

Fasting venous blood samples were collected for the measurement of FFAs, acylated ghrelin, total PYY, leptin, TC, HDL-C, LDL-C, TAG, CRP and IL-6 concentrations. Blood samples were collected from the antecubital vein using a 25 g butterfly needle (BD Vacutainer^®^, Plymouth, UK) whilst participants were in a semi-supine position. Samples were collected into four pre-cooled vacutainers: 10.0 mL EDTA, 4.0 mL EDTA, 5.0 mL SST and 6.0 mL heparin (BD Vacutainer^®^, Plymouth, UK). To prevent the degradation of acylated ghrelin, a 40 μL solution containing potassium phosphate buffer (PBS), p-hydroxymercuribenzoic acid (PHMB) and sodium hydroxide (NaOH) was added immediately to the 4.0 mL EDTA vacutainer which was then centrifuged at 1500× *g* for 10 min at 4 °C. The plasma supernatant was dispensed into a storage tube and 100 µL of 1 M hydrochloric acid was added per millilitre of plasma to preserve acylated ghrelin [22]. Thereafter, samples were spun at 1500× *g* for 5 min at 4 °C prior to storage at −80 °C. The 6.0 mL heparin and 10 mL EDTA vacutainers were centrifuged immediately, while the 5.0 mL SST vacutainer was left at room temperature for 30 min before centrifugation using the same conditions. 

During the OGTT, whole blood was collected using the finger-prick technique into a 20 µL heparin capillary tube (Sanguis Counting, Nümbrecht, Germany) for glucose analysis and into a 300 µL EDTA Microvette tube (Microvette^®^ CB 300 K2E, Sarstedt, Leicester, UK) for insulin analysis. The heparin tube was immediately mixed into a separate 1 mL haemolysing solution and then analysed. The EDTA tube was immediately centrifuged at 1500× *g* for 10 min at 4 °C (Eppendorf^®^ Microcentrifuge 5415R, Eppendorf AG, Hamburg, Germany) and the plasma supernatant was then dispensed into aliquots and stored at −80 °C for later analysis.

### 4.8. Analytical Methods and Biochemical Analysis

#### 4.8.1. LC-MS

LC-MS analysis was carried out using a Dionex 3000 HPLC interfaced with and an Orbitrap Exactive mass spectrometer (Thermo Fisher Scientific, Bremen, Germany). An ACE C4 column (HiChrom, Reading, UK) was used to quantify the free fatty acids. The mobile phase for the elution of the ACE C4 column consisted of 1 mM acetic acid in water (A) and 1 mM acetic acid in acetonitrile (B) at a flow rate of 0.4 mL·min^−1^. The elution gradient was as follows: A:B ratio 40:60 at 0 min, 0:100 at 30 min, 0:100 at 36 min, 40:60 at 37 min and 40:60 at 41 min. 

The nitrogen sheath and auxiliary gas flow rates were maintained at 50 and 17 arbitrary units. The electrospray ionisation (ESI) interface was operated in both positive and negative modes. The spray voltage was 4.5 kV for the positive mode and 4.0 kV for negative mode, while the ion transfer capillary temperature was 275 °C. Full scan data was obtained in the mass-to-charge range of *m*/*z* 75 to *m*/*z* 1200 for both ionisation modes. The MS system was fully calibrated prior to running the samples according to the manufacturer’s guidelines. The resulting data was acquired using the XCalibur 2.1.0 software package (Thermo Fisher Scientific, Bremen, Germany).

#### 4.8.2. LC-MS Sample Preparation and Calibration Series

Aliquots of plasma (0.3 mL) were mixed with 0.2 mL of acetonitrile containing 8 µg mL^−1^ of internal standard. A calibration series was prepared by mixing the diluted fatty acid stock solution with the internal standard to give an internal standard concentration of 4.8 µg mL^−1^ mixed with fatty acid standards in the range 0.8–38.0 µg mL^−1^ (the original standard mixture contained fatty acids at different concentrations: 0.2, 0.4 or 0.6 mg mL^−1^).

#### 4.8.3. Appetite-Related Hormones, Inflammatory and Metabolic Markers

Plasma concentrations of insulin (Mercodia, Uppsala, Sweden), CRP, IL-6 (high sensitivity kit, IBL International, Hamburg, Germany), acylated ghrelin (Bertin Bioreagent, Montigny le Bretonneux, France), total PYY and leptin (Millipore, Billerica, MA, USA) were measured using commercially available enzyme-linked immunosorbent assays. Data for plasma insulin concentrations were analysed in a sub-sample of 8 South Asian and 8 white European participants matched for BMI. Plasma TC, HDL-C, LDL-C and TAG concentrations were determined by enzymatic, colorimetric methods using a bench top analyser (Pentra 400; HORIBA ABX Diagnostics, Montpellier, France). Samples from each participant were analysed in duplicate in the same run to avoid inter-assay variation. Plasma glucose concentrations were analysed immediately in singular using a glucose analyser (Biosen C-Line Clinic, EKF Diagnostic, Barleben, Germany). Coefficients of variation for the assay duplicates were as follows: 3.8% acylated ghrelin, 5.1% total PYY, 4.0% leptin, 4.6% CRP, 8.8% IL-6, 0.4% total cholesterol, 0.9% HDL-C, 0.8% LDL-C, 2.0% TAG and 6.8% insulin.

### 4.9. Data Processing and Statistical Analysis

#### 4.9.1. LC-MS

The Quan Browser in Xcalibur 2.1.0 was used to plot calibration curves (weighted with 1/x) and quantify the responses for the samples against the calibration curves. Then the levels of FFAs in the samples were calculated from the calibration curves by Quan Browser. *p* values and ratios of the mean values for the fatty acids were determined by using Microsoft Excel. The quantitative values for the samples were then mean centred, Pareto scaled and modelled using PCA, OPLS-DA and OPLS with Simca P 14.1 (MKS Umetrics, Umea, Sweden). The variables underpinning both the OPLS-DA and OPLS models were reduced by trial and error in order to produce strong models as indicated by the cross-validation plot for the OPLS-DA model or by the best correlation coefficient for the OPLS models.

#### 4.9.2. Appetite-Related Hormones, Inflammatory and Metabolic Markers

Statistical analyses were conducted using the analytical software SPSS version 23.0 for Windows (SPSS 23.0, IBM Corp, Armonk, NY, USA). The HOMA-IR [48] and insulin sensitivity index [49] were only calculated for the subsample of participants with available insulin data (eight South Asian, eight white European). Normality of the data was checked using Shapiro–Wilk tests. Normally distributed data are presented as mean (SD). Data for appetite-related hormones, inflammatory markers and metabolic markers were not normally distributed and were natural log transformed before analysis. These data are presented as geometric mean (95% confidence interval) and analysis is based on ratios of geometric means and 95% confidence intervals (CI) for ratios. 

Physical and physiological characteristics and dietary intake were compared between the South Asian and white European men using linear mixed models with ethnic group included as a fixed factor. Habitual physical activity levels and sedentary time were compared between ethnic groups using linear mixed models with wear time included as a covariate. The trapezium rule was used to calculate the total area under the curve (AUC) for glucose and insulin during the OGTT. Linear mixed models, both unadjusted and adjusted for percentage body fat, were employed to examine between-group differences in fasting plasma constituents, 2 h glucose and insulin concentrations and AUC values. Differences in glucose and insulin concentrations over the 2 h OGTT were examined using 2 × 5 (group × time) linear mixed models (unadjusted and adjusted for percentage body fat). Absolute standardised effect sizes (ES) (Cohen’s d) were calculated for each variable by dividing the difference between the mean values (South Asian versus white European) with the pooled SD. An ES of 0.2 was considered the minimum important difference, 0.5 moderate and 0.8 large [50]. Etnicity-specific Pearson’s product-moment correlation coefficients (*r*) were used to examine the magnitude of linear relationship between age, body fat percentage, $\dot{V}$O_2max_, sedentary time, MVPA and specific outcome measures including acylated ghrelin, leptin, insulin sensitivity index, CRP and HDL-C in South Asian and white European participants. Statistical significance was accepted as *p* < 0.05.

## 5. Conclusions

The current study provides evidence that levels of circulating FFAs are different between South Asians and white European men. Using an OPLS models, FFAs were positively correlated with body fat percentage and AUC for glucose in South Asian, whereas total step counts were strongly correlated with lower levels of FFAs in white European men. This may suggest that fatty acid metabolism is less responsive to physical activity in South Asian men in comparison to white European men. Healthy South Asian men also exhibited lower concentrations of acylated ghrelin and an adverse CVD and T2D risk marker profile compared with BMI-matched white European men including higher concentrations of insulin, TAG, leptin and CRP, and lower concentrations of HDL-C. Although objectively assessed physical activity levels and sedentary time were similar between the ethnic groups, the lower cardiorespiratory fitness in the South Asian men may contribute to the heightened cardio-metabolic heath risk in this population. Future research that targets the identification of additional parameters of CVD and T2D risk in South Asians should be prioritised. The interpretation of our findings should be tempered by the fact that the number of participants in the study was low.

## Figures and Tables

**Table 1:** Participant characteristics.

	South Asians (*n* = 16)	White Europeans (*n* = 16)	White Europeans vs. South Asians 95% CI *^a^*	Effect Size
Age (years)	30 (8)	36 (8)	−11 to 0.04	0.72
Stature (cm)	176.3 (6.9)	179.3 (4.7)	−7.2 to 1.3	0.49
Body mass (kg)	79.4 (14.6)	80.5 (8.4)	−9.7 to 7.5	0.09
Body mass index (kg·m^−2^)	25.7 (5.2)	25.2 (3.3)	−2.6 to 3.7	0.12
Fat-free mass (kg)	57.4 (5.3)	64.5 (5.7)	−11.2 to −3.2 *	1.29
Fat mass (kg)	22.1 (11.0)	16.0 (6.9)	−0.5 to 12.7 *	0.66
Body fat (%)	26.4 (9.0)	19.5 (7.0)	1.1 to 12.8 *	0.86
Waist Circumference (cm)	87.8 (13.4)	85.5 (6.6)	−5.4 to 9.9	0.21
Resting sBP (mmHg)	120 (11)	122 (10)	−10 to 5	0.23
Resting dBP (mmHg)	78 (8)	77 (11)	−6 to 7	0.05
$\dot{V}$O_2max_ (L·min^−1^)	2.97 (0.55)	4.00 (0.60)	−1.44 to −0.61 *	1.79
$\dot{V}$O_2max_ (mL·kg^−1^·min^−1^)	38 (9)	50 (8)	−18 to −5 *	1.36

All values are mean (SD). Data were analysed using linear mixed models; sBP, systolic blood pressure; dBP, diastolic blood pressure; $\dot{V}$O_2max_, maximum oxygen uptake; *^a^* 95% confidence interval of the mean absolute difference between groups; * Significant difference between South Asians and white Europeans (*p* < 0.05).

**Table 2:** Concentration of 19 FFAs in plasma from South Asian and white European men.

Free Fatty Acid	South Asians (*n* = 16)	White Europeans (*n* = 16)	RT (min)	*m*/*z*	*p* Value SA/WE	Ratio of Mean Conc. SA/WE
Laurate	29.8 (12.2)	28.3 (14.5)	8.3	199.169	0.040	1.092
Myristate	48.0 (44.8)	33.5 (34.3)	11.5	227.200	0.011	1.478
Pentadecenoate	12.5 (7.9)	12.3 (4.5)	10.9	239.200	0.224	1.032
Pentadecanoic	13.5 (21.5)	12.5 (26.7)	13.3	241.216	0.214	1.111
Palmitoleic	51.3 (66.8)	36.3 (60.5)	12.4	253.216	0.137	1.371
Palmitate	81.0 (33.3)	54.8 (30.6)	15.2	255.232	0.004	1.487
Linolenic	17.8 (30.1)	13.8 (35.0)	11.6	277.216	0.017	1.328
Linoleate	322.3 (31.2)	206.0 (44.7)	13.6	279.232	0.005	1.621
Oleate	633.8 (43.4)	473.5 (42.5)	16.1	281.247	0.081	1.317
Stearic	193.5 (29.8)	155.3 (28.6)	19.2	283.263	0.095	1.227
Eicosapentaenoic	9.3 (19.7)	10.3 (37.9)	11.5	301.216	0.449	0.899
Eicosatetraenoic	25 (30.9)	25.3 (35.9)	13.4	303.232	0.958	0.972
Eicosatrienoic	7.0 (27.2)	6.5 (27.0)	14.8	305.247	0.554	1.059
Eicosadienoic	12.2 (12.3)	11.5 (14.7)	17.1	307.263	0.215	1.068
Eicosenoic	14.0 (24.9)	13.3 (37.6)	19.8	309.279	0.666	1.022
Arachidate	35.3 (59.2)	49.0 (53.4)	23.1	311.294	0.074	0.681
Docosahexaenoic	18.3 (43.0)	25.3 (69.9)	13.1	327.232	0.100	0.683
Tricosanoate	20.0 (53.3)	16.5 (47.2)	28.1	353.341	0.456	1.179
Lignocerate	13.4 (49.5)	10.8 (29.1)	29.6	367.357	0.250	1.225

All values are mean (RSD %); RT, retention time; *m*/*z*, mass-to-charge ratio; * Significant difference between South Asians and white Europeans (*p* < 0.05).

**Table 3:** Fasting plasma concentrations in South Asian and white European men.

	South Asians (*n* = 16)	White Europeans (*n* = 16)	Model 1 *^a^*		Model 2 *^b^*	
			White Europeans vs. South Asians 95% CI *^c^*	Effect Size	White Europeans vs. South Asians 95% CI *^c^*	Effect Size
Acylated ghrelin (pg·mL^−1^)	35.7 (25.8 to 49.4)	67.7 (48.8 to 93.7)	−67 to −16% *	1.00	−59 to 5%	0.65
Total peptide YY (pg·mL^−1^)	90.3 (80.0 to 101.8)	83.8 (74.3 to 94.5)	−9 to 28%	0.31	−9 to 33%	0.39
Leptin (ng·mL^−1^)	6.11 (3.76 to 9.92)	2.13 (1.31 to 3.46)	44 to 470% *	1.11	−1 to 91%	0.33
C-reactive protein (µg·mL^−1^)	0.89 (0.57 to 1.38)	0.42 (0.27 to 0.65)	14 to 298% *	0.87	−17 to 197%	0.52
Interleukin-6 (pg·mL^−1^) *^d^*	0.71 (0.49 to 1.03)	0.45 (0.32 to 0.64)	−5 to 161%	0.86	−19 to 119%	0.54
TC (mmol·L^−1^)	4.37 (4.04 to 4.73)	4.28 (3.96 to 4.63)	−9 to 14%	0.13	−11 to 14%	0.03
HDL-C (mmol·L^−1^)	1.10 (0.98 to 1.24)	1.32 (1.17 to 1.48)	−29 to −1% *	0.78	−22 to 8%	0.36
TC/HDL-C ratio	3.97 (3.50 to 4.51)	3.25 (2.86 to 3.69)	2 to 47% *	0.80	−8 to 30%	0.35
LDL-C (mmol·L^−1^)	2.72 (2.42 to 3.06)	2.45 (2.18 to 2.76)	−6 to 31%	0.45	−12 to 25%	0.20
Triacylglycerol (mmol·L^−1^)	1.16 (0.91 to 1.48)	0.81 (0.63 to 1.03)	1 to 102% *	0.74	−18 to 48%	0.20

All values are geometric mean (95% confidence interval). Statistical analyses are based on log-transformed data. Data were analysed using linear mixed models; TC, total cholesterol; HDL-C, high-density lipoprotein cholesterol; LDL-C, low-density lipoprotein cholesterol; *^a^* Model 1: unadjusted; *^b^* Model 2: adjusted for body fat percentage; *^c^* 95% confidence interval for the ratio of geometric means; *^d^* Data for interleukin-6 available for *n* = 9 South Asian and *n* = 10 white European; * Significant difference between South Asians and white Europeans (*p* < 0.05).

**Table 4:** Plasma glucose and insulin concentrations during the OGTT in South Asian and white European men.

	South Asians (*n* = 16)	White Europeans (*n* = 16)	Model 1 *^a^*		Model 2 *^b^*	
			White Europeans vs. South Asians 95% CI *^c^*	Effect Size	White Europeans vs. South Asians 95% CI *^c^*	Effect Size
**Glucose**						
Fasted (mmol·L^−1^)	4.50 (4.28 to 4.74)	4.71 (4.47 to 4.96)	−11 to 3%	0.45	−12 to 3%	0.51
2 h (mmol·L^−1^)	4.78 (4.11 to 5.56)	4.16 (3.58 to 4.83)	−7 to 42%	0.47	−14 to 35%	0.25
**Insulin**						
Fasted (µU·mL^−1^)	7.21 (4.90 to 10.60)	4.22 (2.87 to 6.20)	−1 to 195%	1.06	−18 to 118%	0.58
2 h (µU·mL^−1^)	23.49 (10.32 to 53.49)	5.84 (2.56 to 13.29)	26 to 1188% *	1.28	−10 to 391%	0.68
**HOMA-IR**	1.50 (1.00 to 2.26)	0.91 (0.60 to 1.36)	−7 to 195%	0.94	−23 to 109%	0.44
**Insulin sensitivity index**	5.62 (3.32 to 9.49)	14.41 (8.53 to 24.36)	−81 to −18% *	1.22	−65 to 1%	0.68

All values are geometric mean (95% confidence interval) for *n* = 32 (glucose) and *n* = 16 (insulin, HOMA-IR, insulin sensitivity index). Statistical analyses are based on log-transformed data. Data were analysed using linear mixed models; OGTT, oral glucose tolerance test; HOMA-IR, homeostasis model assessment of insulin resistance; *^a^* Model 1: unadjusted; *^b^* Model 2: adjusted for body fat percentage; *^c^* 95% confidence interval for the ratio of geometric means; * Significant difference between South Asians and white Europeans (*p* < 0.05).

## Supplementary Materials

The following are available online at https://www.mdpi.com/2218-1989/9/4/71/s1. Table S1: Habitual physical activity levels and sedentary time in South Asian and white European men. Table S2: Average daily energy, macronutrient and micronutrient intakes in South Asian and white European men. Table S3: Ethnicity-specific Pearson’s correlation coefficients between the various predictors and metabolic risk markers. Figure S1: Chromatogram for the FFAs detected in plasma compared with a chromatogram for the calibration standards (concentration range 3.2–9.6 µg/mL). Figure S2: Cross-validation plot corresponding to figure 2. Figure S3: Loadings plot corresponding to the OPLS-DA plot shown Figure 2. Figure S4: OPLS plot of predicted BMI against actual BMI for South Asian participants. Figure S5: Loadings plot for Figure 3. Figure S6: Extracted ion traces for two markers of higher body fat percentage, palmitic acid and eicosapentaenoic acid. The levels of the acids are highest in subject P31 who had the highest body fat percentage and lowest in participant P8 who had the lowest body fat percentage. Figure S7: Loadings plot for Figure 7. Figure S8: Extracted ion traces for palmitoleic acid and linoleic acid for participant P24 who has the lowest step count and participant P14 who has the highest step count.
