# Supplementary material for: Plasma Free Fatty Acids Metabolic Profile with LC-MS and Appetite-Related Hormones in South Asian and White European Men in Relation to Adiposity, Physical Activity and Cardiorespiratory Fitness: A Cross-Sectional Study
Source: Metabolites. 2019 Apr 13;9(4):71. doi: 10.3390/metabo9040071 (PMC6523813; doi:10.3390/metabo9040071)
Supplement: Supplementary file 1 [file metabolites-09-00071-s001.pdf]

metabolites

Plasma Free Fatty Acids Metabolic Profile with LC-MS and Appetite-Related Hormones in South Asian and White European men in Relation to Adiposity, Physical Activity and Cardiorespiratory Fitness: A Cross-Sectional Study

Simone Benedetti[1], Naser F. Al-Tannak[2], Mansour Alzharani[3], Hannah J. Moir[1], David J. Stensel[4,5],

Alice E. Thackray[4,5], Declan Naughton[1], Mehmet T. Dorak[1], Owen Spendiff[1], Natasha Hill[1], David

G. Watson[3,*] and Judith Allgrove[1,*]

[1] Applied & Human Sciences, School of Life Sciences, Pharmacy & Chemistry, Kingston University London, Kingston upon Thames KT1 2EE, UK; S.Benedetti@kingston.ac.uk (S.B); H.Moir@kingston.ac.uk (H.J.M.); D.Naughton@kingston.ac.uk (D.N); M.Dorak@kingston.ac.uk (M.T.D); O.Spendiff@kingston.ac.uk (O.S); N.Hill@kingston.ac.uk (N.H);

[2] Department of Pharmaceutical Chemistry, Faculty of Pharmacy, Kuwait University, P.O. Box 23924, Safat, 13110 Kuwait City, Kuwait; dr_altannak@HSC.EDU.KW (N.F.AT);

[3] Strathclyde Institute of Pharmacy and Biomedical Sciences, University of Strathclyde, The John Arbuthnott Building, 161 Cathedral Street, Glasgow G4 0RE, UK; mansour.alzahrani@strath.ac.uk (MA);

[4] National Centre for Sport and Exercise Medicine, School of Sport, Exercise and Health Sciences, Loughborough University, Loughborough, LE11 3TU, UK; D.J.Stensel@lboro.ac.uk (D.J.S); A.E.Thackray@lboro.ac.uk (A.E.T).

[5] University Hospitals of Leicester NHS Trust, Infirmary Square, Leicester LE1 5WW, UK; D.J.Stensel@lboro.ac.uk (D.J.S); A.E.Thackray@lboro.ac.uk (A.E.T).

[*] Correspondence: d.g.watson@strath.ac.uk; Tel.: +44-141-54-82-651;

J.Allgrove@kingston.ac.uk; Tel: +44-208-41-77-360.

Table S1. Habitual physical activity levels and sedentary time in South Asian and White European men.

	**South Asians (n = 13)**	**White Europeans (n = 13)**	**White Europeans vs. South Asians 95% CI[a]**	**Effect size**
Wear time (min·day^{-1})	828 (87)	858 (64)	–92 to 32	0.39
Total activity (counts·min^{-1}·day^{-1})	396 (115)	474 (124)	–182 to 19	0.65
Sedentary time (min·day^{-1})	546 (82)	528 (66)	–16 to 87	0.25
Light physical activity (min·day^{-1})	172 (73)	197 (47)	–62 to 32	0.41
MVPA (min·day^{-1})	53 (15)	57 (24)	–21 to 14	0.18
Total steps (per day)	8759 (1935)	10157 (1981)	–2725 to 417	0.71

All values are mean (SD). Data were analysed using linear mixed models. Models for total activity, sedentary time, light physical activity, MVPA and total steps included wear time as a covariate.*MVPA* moderate-to-vigorous physical activity; [a] 95% confidence interval of the mean absolute difference between groups.

Table S2. Average daily energy, macronutrient and micronutrient intakes in South Asian and White European men.

	**South Asians (n=16)**	**White Europeans (n=16)**	**White Europeans vs. South Asians 95% CI[a]**	**Effect size**
Energy (kcal·day^{-1})	2136 (527)	2060 (603)	–333 to 486	0.12
Carbohydrate (g·day^{-1})	252 (75)	217 (105)	–31 to 100	0.38
Fat (g·day^{-1})	81.5 (30.9)	77.4 (37.5)	–20.6 to 29.0	0.12
Protein (g·day^{-1})	98.7 (28.5)	123.7 (46.8)	–53.0 to 3.1	0.64
% energy from carbohydrate	48 (19)	41 (10)	–0.5 to 13	0.65
% energy from fat	34 (8)	33 (12)	–7 to 8	0.02
% energy from protein	19 (6)	26 (13)	–14 to 0.4	0.70
Calcium (mg·day^{-1})	723 (470)	691 (311)	–256 to 319	0.08
Magnesium (mg·day^{-1})	262 (113)	311 (81)	–120 to 22	0.50
Sodium (mg·day^{-1})	1976 (1066)	2154 (884)	–885 to 529	0.18
Folate (µg·day^{-1})	256 (142)	229 (66)	–53 to 107	0.24
Vitamin D (µg·day^{-1})	2.2 (1.9)	5.9 (8.1)	–8.0 to 0.5	0.63
Vitamin C (mg·day^{-1})	116.3 (83.0)	132.4 (169.9)	–112.7 to 80.4	0.12

All values are mean (SD). Data were analysed using linear mixed models. Energy, macronutrient and micronutrient values were recorded for three days (two weekdays and one weekend).

[a] 95% confidence interval of the mean absolute difference between groups.

Table S3. Ethnicity-specific Pearson's correlation coefficients between the various predictors and metabolic risk markers.

	Acylated ghrelin[a]		Leptin		Insulin sensitivity index		C-reactive protein[a]		HDL-C[a]	
	**South Asian**	**White European**	**South Asian**	**White European**	**South Asian**	**White European**	**South Asian**	**White European**	**South Asian**	**White European**
Age	0.05	0.40	0.07	–0.03	0.11	–0.14	0.45	–0.01	–0.09	0.38
Body fat	–0.25	**–0.73***	**0.88****	**0.84****	**–0.83***	**–0.76***	0.52	0.16	–0.28	**–0.81***
$\dot{V}O_2$ max	0.37	0.27	–0.01	–0.12	0.28	**0.80***	0.29	0.38	–0.06	0.24
Sedentary time	0.20	–0.13	0.47	0.05	0.04	–0.27	0.50	0.11	–0.06	–0.19
MVPA	0.05	0.04	–0.18	–0.50	0.14	0.42	–0.43	–0.02	0.39	0.18

[a] Statistical analyses are based on log-transformed data.

HDL-C, high-density lipoprotein cholesterol; *VO_2 max,* maximum oxygen uptake; *MVPA* moderate-to-vigorous physical activity

* $p < 0.05$; ** $p < 0.001$

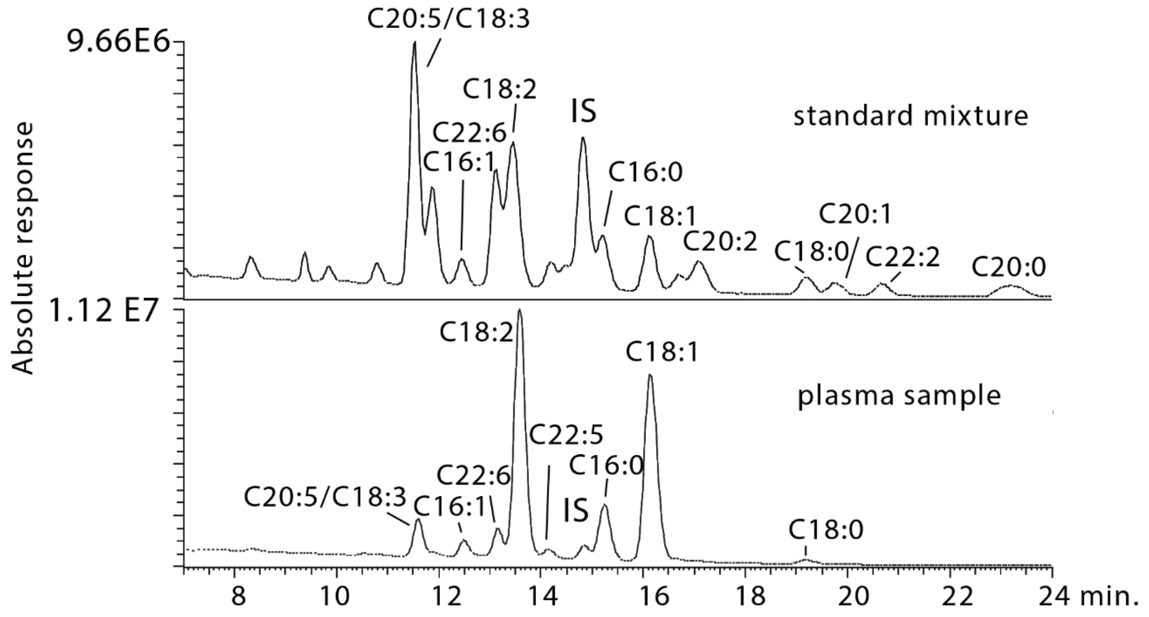

Figure S1. Chromatogram for the fatty acids detected in plasma compared with a chromatogram for the calibration standards (concentration range 3.2–9.6 μg·mL^{-1}).

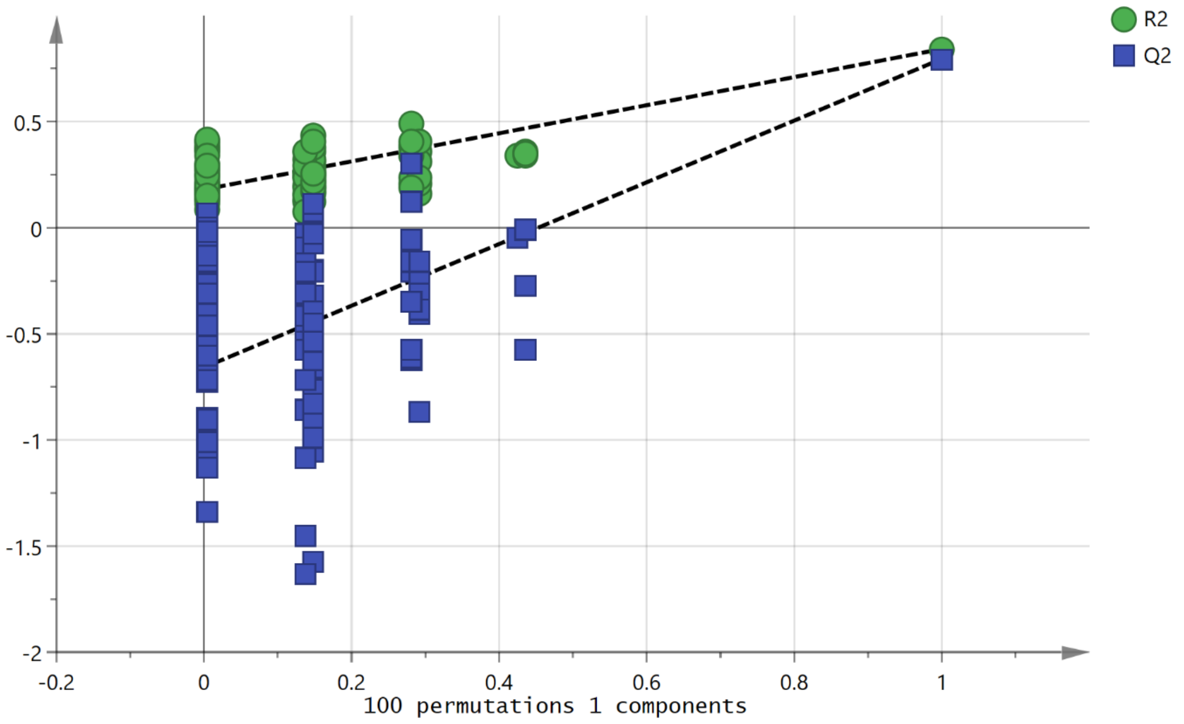

Figure S2. Cross-validation plot corresponding to Figure 2.

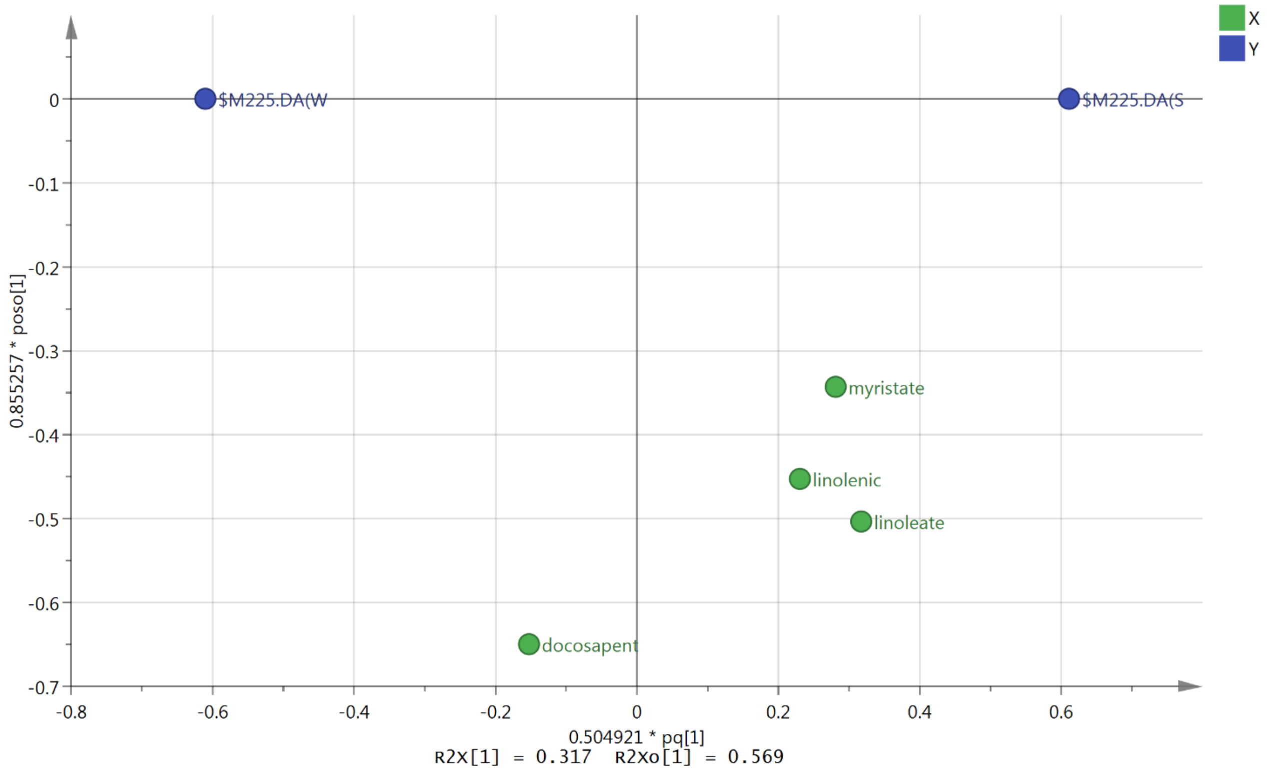

Figure S3. Loadings plot corresponding to the OPLS-DA plot shown Figure 2.

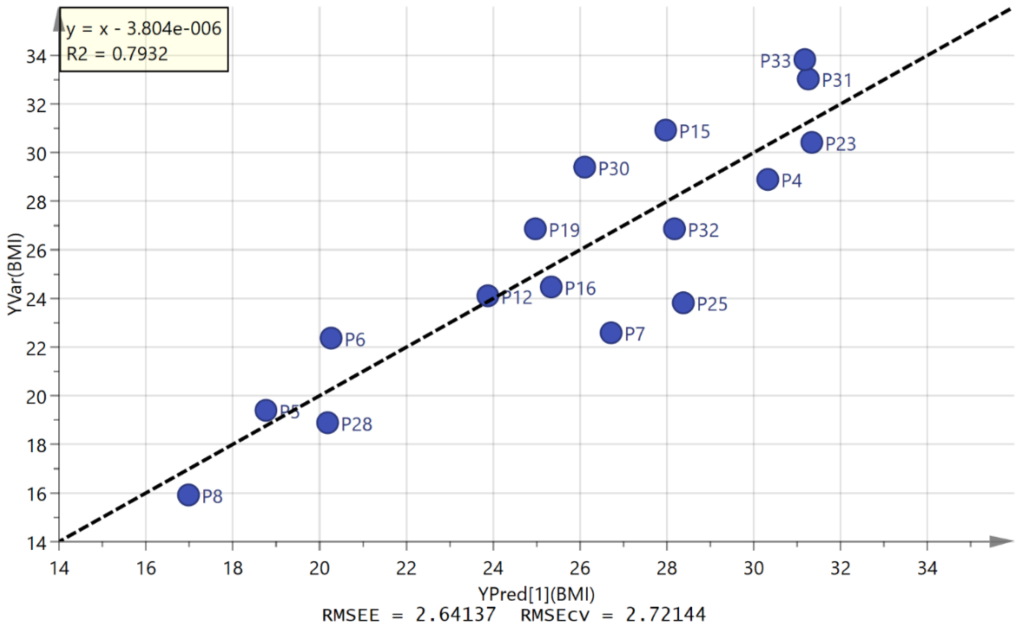

Figure S4. OPLS plot of predicted BMI against actual BMI for South Asian men.

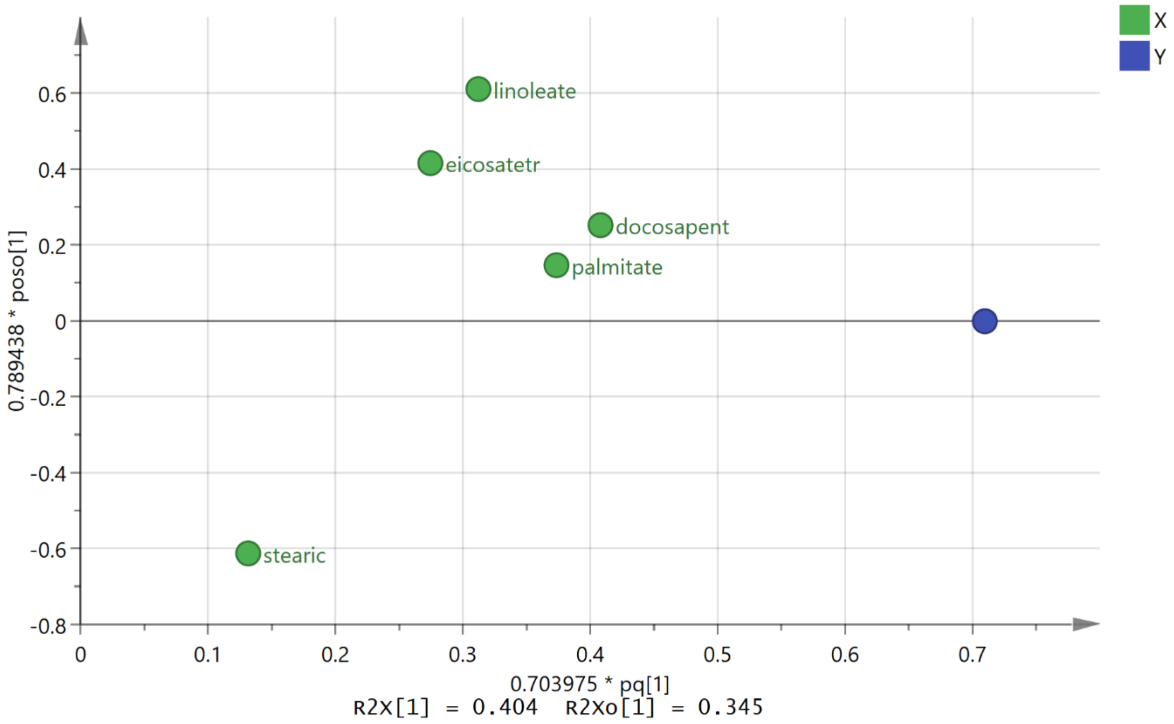

Figure S5. Loadings plot for Figure 3.

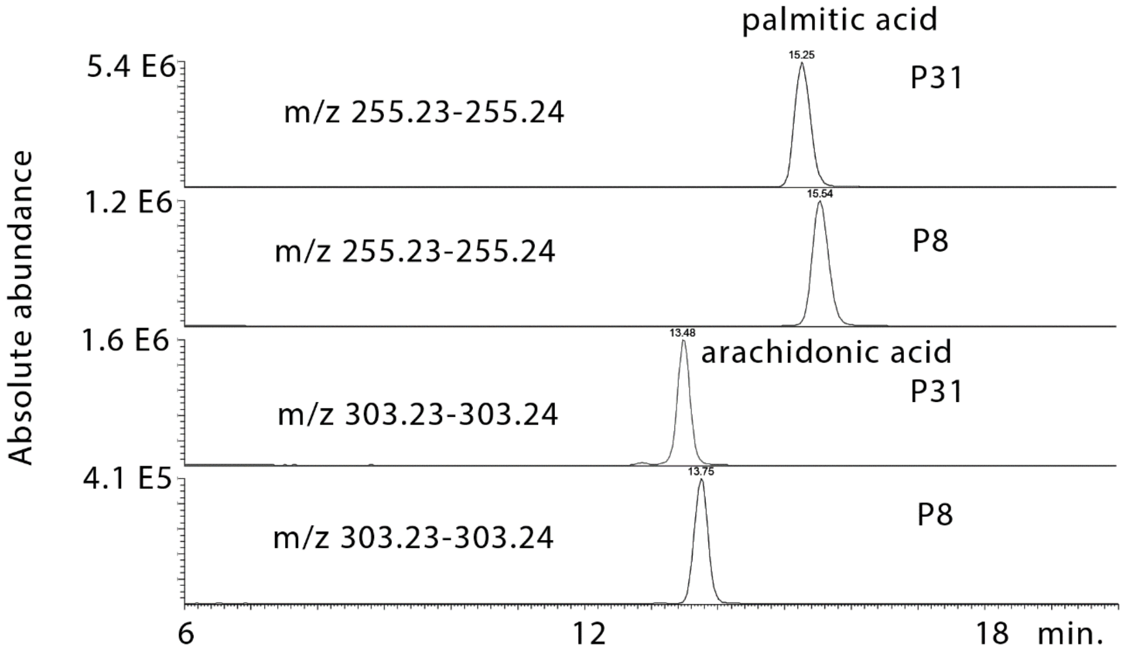

Figure S6. Extracted ion traces for two markers of higher body fat percentage, palmitic acid and eicosapentaenoic acid. The levels of the acids are highest in participant P31 (South Asian) who had the highest body fat percentage and lowest in participant P8 (South Asian) who had the lowest body fat percentage.

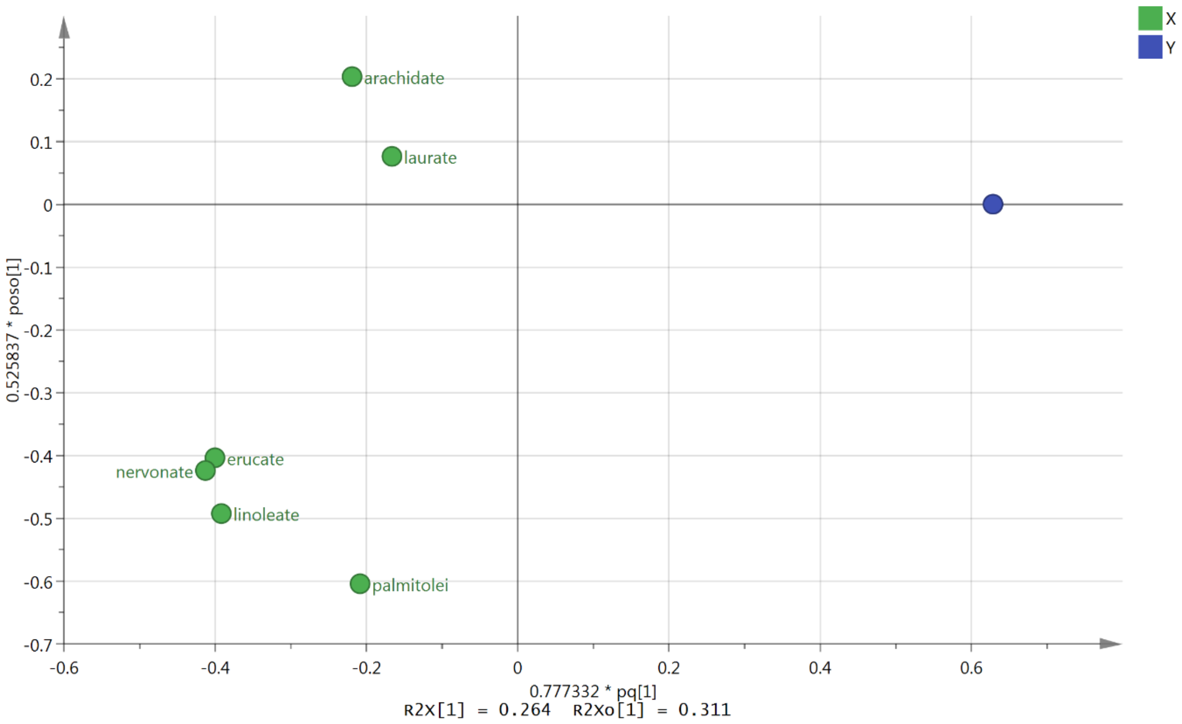

Figure S7 Loadings plot for Figure 7.

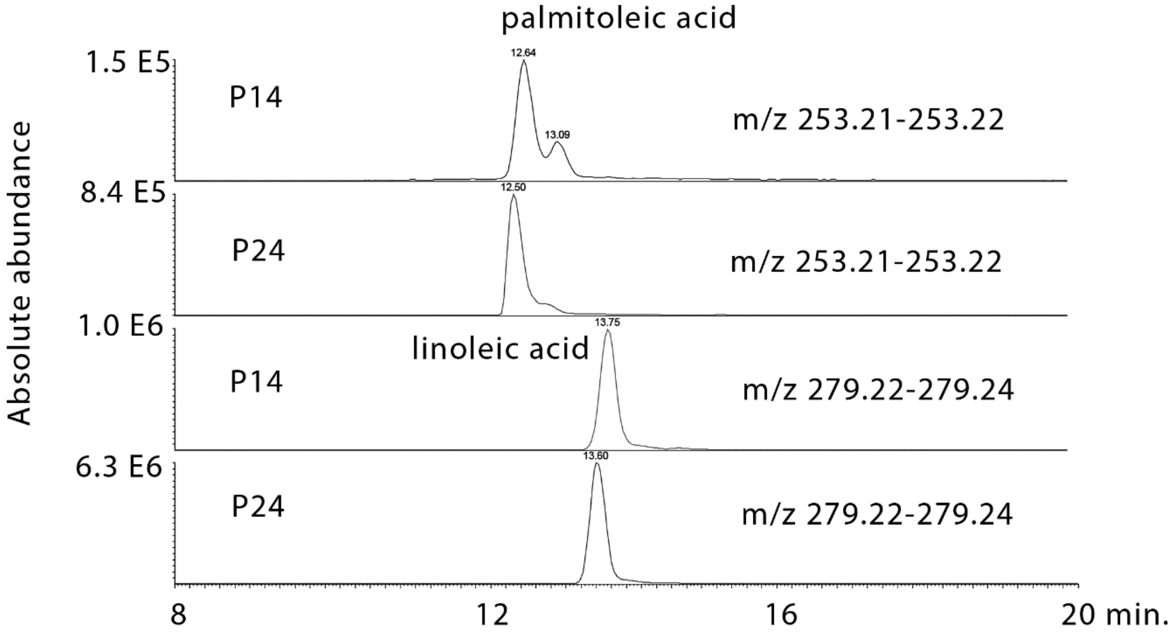

Figure S8 Extracted ion traces for palmitoleic acid and linoleic acid for participant P24 (White European) who had the lowest step count and participant P14 (white European) who had the highest total step counts.
